# Dexamethasone disrupts intercellular junction formation and cytoskeleton organization in human trabecular meshwork cells

**Published:** 2010-01-16

**Authors:** Ye Hong Zhuo, Yuan He, Kar Wah Leung, Fei Hou, Yi Qing Li, Fang Chai, Jian Ge

**Affiliations:** 1 State Key Laboratory of Ophthalmology, Zhongshan Ophthalmic Center, Sun Yat-sen University, Guangzhou, China; 2 Department of Ophthalmology in the Second Affiliated Hospital of Xi’an College of Medicine, Xi’an, China; 3 Department of Biology, Hong Kong University of Science and Technology; 4 Shenzhen Eye Hospital, Shenzhen, China

## Abstract

**Purpose:**

Patients reproduce symptoms of primary open-angle glaucoma (POAG) when treated with glucocorticoids (GCs) topically on the eyes. Here we investigated the effects of GCs on junctional protein expression and cytoskeleton organization in primary human trabecular meshwork (TM) cultures to understand the molecular pathologies of POAG.

**Methods:**

Human TM cells from POAG (GTM) and age-matched nondiseased (NTM) individuals were obtained by standard surgical trabeculectomy. Some of the cultures were treated with dexamethasone (DEX), a synthetic GC, at 1–5×10^−7^ mol/l for 1–7 days. The expression levels of zonula occluden-1 (*ZO-1*) and connexin43 (*Cx43*) in TM cells with or without DEX treatment were measured using reverse transcription (RT)–PCR, immunocytochemistry, and western blot analysis.

**Results:**

mRNA and proteins of *ZO-1* and *Cx43* were found in both NTM and GTM cells. ZO-1 and Cx43 were located on the plasma membrane, especially along the border of adjacent cells. ZO-1 had no marked changes in localization in NTM and GTM cells after treatment with 10^−7^ mol/l DEX for 48 h, whereas Cx43 appeared to increase in the cytoplasm. mRNA of two *ZO-1* isoforms, α+ and α–, were present in TM cells, and the former was expressed less than the latter. Only ZO-1 α– isoform protein was expressed in NTM cells, whereas proteins of both isoforms were found in GTM cells. DEX increased the protein levels of ZO-1 and Cx43 in both NTM and GTM cells. DEX also altered the F-actin architecture and promoted cross-linked actin network formation, the effects of which were more pronounced in GTM cells.

**Conclusions:**

Our findings not only provide molecular insights to the pathogenesis of GC-induced glaucoma but also suggest that junctional proteins ZO-1 and Cx43 as well as F-actin are targets for developing new modalities in glaucoma therapy.

## Introduction

Dexamethasone (DEX), a synthetic glucocorticoid (GC), is a potent and effective ocular anti-inﬂammatory agent that is topically applied in ocular conditions, such as keratitis, uveitis, and iritis [1]. However, the adverse effects of prolonged use of DEX include decreased aqueous humor outflow and increased intraocular pressure (IOP), which may cause the onset of secondary glaucoma. The exact molecular mechanism of glucocorticoid-induced glaucoma (GIG) is still elusive, but evidence points to excessive extracellular matrix (ECM) material aggregation within the outﬂow channels in trabecular meshwork (TM) tissues as a result of ECM degradation inhibition, which subsequently leads to increased outﬂow resistance [2-4]. GC-induced ocular hypertension shares some clinical features with primary open-angle glaucoma (POAG). Besides IOP elevation, both secondary and primary glaucoma have selective retinal ganglion cell death that causes visual field changes, nerve fiber layer defects, and eventual irreversible blindness [4,5]. Several studies have noted that the GC-induced changes in TM can partially reflect the pathological mechanisms of POAG [6]. Investigations into the molecular mechanisms of GIG may provide new insights into the pathology of POAG. Here we use DEX-treated TM cells to investigate the molecular changes in TM cells obtained from nondiseased individuals and POAG patients.

TM regulates the drainage rate by changing the intercellular space through a combination of actions. Other than ECM turnover rate regulation, cellular contractility and cellular volume are partly controlled by cytoskeleton and junctional proteins. F-actin, a major component of cytoskeleton, is organized to respond to cell contraction and to participate in generating forces responsible for continued development and maintenance of tension [7]. Contraction of the TM reduces the intercellular spaces and thus reduces aqueous humor outflow [8]. A previous study showed that DEX induces F-actin expression and enhances fibroblast-mediated contraction [9]. In addition, actin becomes tangled and dysorganized in the TM and Schlemm’s canal of glaucomatous eyes or in DEX-treated cultures, in which these cells are more resistant to fluid outflow [10,11]. Actin cytoskeleton re-organization also alters cell–ECM interaction.

The presence of tight junctions and gap junctions has been demonstrated in TM cells using freeze-fracture techniques [12-15]. Junctional-associated proteins zonula occluden-1 (ZO-1) and connexin43 (Cx43) are thought to be closely related to the fluid flow resistance [2,16]. F-actin interacts with ZO-1 to help intercellular tight junction assembly [17,18], in which the tightness and distribution of the tight junctions influence the aqueous humor outflow rate [13]. In addition, ZO-1 complexes with Cx43, a gap junction protein [19], in which Cx43 is required for production of the aqueous humor [20]. Since mutation of myocilin leads to the early onset of glaucoma, we speculate that mutation of *ZO-1*, a gene located on the same chromosome region as myocilin (15q), may also lead to the pathology of glaucoma.

Molecular alterations of TM may affect the outflow facility and subsequently lead to pathogenesis of GIG and POAG. In this study, we therefore focus on investigating how glaucomatous conditions or DEX alters F-actin, ZO-1, and Cx43 in TM cells. We observed that both nondiseased trabecular meshwork (NTM) cells and human TM cells from individuals with POAG (GTM cells) express the ZO-1 α– isoform, while the α+ isoform is unique to GTM cells, indicating the possible involvement of the α+ isoform in transendothelial outflow resistance. DEX increases expression of the tight junction-associated protein ZO-1 and the gap junction protein Cx43 in both NTM and GTM cells. DEX also aggravates actin cytoskeleton dysorganization and cross-linked actin network (CLAN) formation in GTM cells.

## Methods

### Chemical reagents

All tissue culture reagents were obtained from Gibco BRL (Gaithersburg, MD). DEX was purchased from Sigma (St. Louis, MO). Mouse anti-ZO-1, anti-Cx43, and anti-vinculin were purchased from Zymed Laboratories (San Francisco, CA). Actin cytoskeleton and the Focal Adhesion Staining kit were purchased from Chemicon International, Inc. (Nutley, NJ).

### Tissue procurement and cell culture

We followed the standard examination and tissue collection procedures [21,22]. Prior to the surgery, clinical data were collected for each patient, including age, gender, use of prostaglandin analogues, number of argon laser trabeculoplasties and other ocular surgical interventions, type and duration of glaucoma, IOP, and visual acuity. Glaucoma diagnosis was based on careful clinical eye examination, including slit lamp, optical coherence topography, gonioscopy, fundus photography, and visual field. All patients underwent slit lamp examination again the day before surgery.

Normal human eyes were obtained from the Zhongshan Ophthalmic Center Eye Bank in Guangzhou, China [21,22]. The procurement of tissue was approved by the Institutional Review Board Committee at the Sun Yat-sen University at Guangzhou, China. Normal TM cells were collected from eight post-mortem non-diseased human donor eyes within 24 h of death. The ages of the donors ranged from 20 to 60 years and the gender was male. After written informed consent, TM specimens from eight POAG patients (15–60 years, 8 males) recruited in the Eye Hospital, Zhongshan Ophthalmic Center, were obtained within 1 h after standard surgical trabeculectomy for therapeutic purposes. The TM tissues for the POAG samples were obtained from individuals with a similar stage of glaucoma after diagnosis by glaucoma specialists. These patients received the prostaglandin analogs, latanoprost (0.005%) and travoprost (0.004%) for similar lengths of time. None of the individuals from which the TM samples were obtained received steroid medications previously. The average duration of glaucoma for the POAG patients was approximately 2 years and none had a record of systemic disease. Tissue from each patient was used to generate primary cultures of TM cells, as described.

The TM tissue of each of the non-diseased donors and POAG patients were used to generate an independent primary culture of TM cells. The TM cells derived from the non-diseased donors were used as controls in the following experiments of this study. The samples were not pooled at any time in these experiments. Primary cultures were used at passage three to six for each experiment. Each study was performed three times, and each trial contained three measurements of each sample. The average measurements from these studies were used to generate the data.

Briefly, the human TM was carefully dissected from the anterior segments and the whole corneal layer of the human donor eyes. The explants were placed in 24-well culture plates (Corning Costar, Cambridge, MA) containing Dulbecco’s modified Eagle’s medium,  which was supplemented with 15% fetal bovine serum, 2 mmol/l L-glutamine, penicillin (100 U/ml), and streptomycin (100 μg/ml). Cells from the TM migrated from the explants in approximately 7 days and formed a confluent monolayer 2–5 days later. Second- or third-passage cells were used for all the studies described here. Cells obtained from age-matched NTM cells and GTM cells were seeded at a density of 1×10^5^ cells/well, using 6-well tissue culture plates (Corning Costar, Cambridge, MA). Micrographs of the cultures were taken 3 days post seeding, at approximately 80% confluency.

As described in our previous study [22], the expression of fibronectin (FN), laminin (LN), and neuron-specific enolase (NSE) was used to examine the primary TM cultures established from normal and POAG individuals indeed contain TM cells. Briefly, immunolabeling studies were carried out on ice-cold 4% paraformaldehyde fixed TM cells. After permeabilization using Triton X-100, block using bovine serum albumin (BSA), and quench endogenous peroxidase activity with 3% hydrogen peroxide (H_2_O_2_), the cells were immunolabeled with mouse monoclonal anti-FN, rabbit polyclonal anti-LN, or mouse monoclonal anti-NSE. All primary antibodies were obtained from Santa Cruz Biotechnology (Santa Cruz, CA). After washes, the cells were then incubated with biotinylated goat anti-mouse or goat anti-rabbit IgG (Vector Laboratories), before reaction with the avidin-biotin-peroxidase complex. We also incubated the cells with the secondary antibody alone as negative control. After a series of washes, the specimens were treated with 3,3'-diaminobenzidine (DAB)/peroxidase reaction (Vector DAB substrate kit; Vector Laboratories), washed, treated with hematoxylin counterstain, washed again, and then dried at room temperature. The samples were then dehydrated in a graded series of alcohols and cover slipped with 1, 3-diethyl-8-phenylxanthine (DPX). The staining pattern for each antibody was visualized using a phase-contrast microscope (Leica, DM IRB, Germany).

### Dexamethasone treatments

Stock solutions of 1 mM DEX was dissolved in 95% ethanol and stored in 4 °C. NTM and GTM cells were treated with DEX as mentioned below. All TM cells were divided into control and treatment groups. Treatment groups were grown in media containing 1×10^−7^ mol/l DEX to examine the change in expression of ZO-1 and Cx43 and in 5×10^−7^ mol/l DEX to examine the organization of the F-actin cytoskeleton. The control group was grown in normal media and received equivalent volumes of ethanol. Morphological changes in the primary cultures were examined by light microscopy.

### Analysis of *ZO-1* and *Cx43* expression

*ZO-1* and *Cx43* mRNA expression were analyzed by reverse transcription (RT)–PCR. Total RNA from NTM and GTM cells was isolated using commercially available RNeasy kit (Qiagen, Valencia, CA). Briefly, 2–10×10^6^ cells/sample were lysed and eluted through a mini spin column to enrich the RNA content. After partially purified RNA was treated with DNase to remove contaminating genomic DNA. First strand cDNA was synthesized using the iScript cDNA synthesis kit (BioRad, Hercules, CA). RT–PCR was performed using iTaq polymerase (BioRad) at an annealing temperature of 55 °C for 35 cycles for *ZO-1*, *Cx43*, and glyceraldehyde-3-phosphate dehydrogenase (*GAPDH*) primers. The PCR was harvested during the linear part of the amplification increase. The primer sequences for *ZO-1* and *Cx43* are (sense) 5′-GCA GCC ACA ACC AAT TCA TAG-3′ and (antisense) 5′-GCA GAC GAT GTT CAT AGT TTC G-3′; and (sense) 5′-CAA TCA CTT GGC GTG ACT TC-3′ and (antisense) 5′-GTT TGG GCA ACC TTG AGT TC-3′, respectively. The *ZO-1* primers detect both α+ (amplicon size=529 bp) and α– (amplicon size=290 bp) isoforms. *GAPDH* was used as the internal RNA loading control, and samples where no reverse transcriptase was added to the PCR experiments were used as negative controls to confirm that amplification was RNA dependent. PCR products were resolved by 1.5% agarose gel electrophoresis.

For western blot analysis, NTM and GTM cells treated with or without DEX were lysed using cytobuster lysis buffer (Novagen, Madison, WI), and protein concentrations in the supernatant were estimated using the Dc Protein Assay kit (BioRad). Protein (30 μg) was separated by SDS–PAGE and transferred onto nitrocellulose membranes (BioRad). After blocking with 5% (w/v) nonfat dried milk, membranes were incubated with primary antibodies (anti-ZO-1, 1:500 or anti-Cx43, 1:1,000) overnight at 4 °C, followed by washes and incubation with horseradish peroxidase (HRP)-conjugated secondary antibodies for 1 h at room temperature. Bound antibody was determined using the Bio-Rad electrochemiluminescence detection system.

### F-actin imaging in trabecular meshwork cells

Phalloidin binds specifically to the F-actin polymer in mammalian cells and was used to visualize the organization of F-actin in TM cells [23]. Cells seeded on polylysine (10 μg/ml)-coated glass chamber slides at a density of 2,000 cells/chamber were washed, fixed in ice-cold 4% paraformaldehyde for 15 min, and permeabilized in 100 mM phosphate buffer containing 1 mg/ml bovine serum albumin, and 0.2% Triton X-100 for 4 min. After quenching the endogenous peroxidase activity with 3% H_2_O_2_, the cells were incubated with 0.5% blocking reagent for 30 min (TSA-Direct kit; Dupont-NEN, Boston, MA). The cells were then immunolabeled with anti-vinculin (1:200) at room temperature for 1 h. Normal mouse immunoglobulin G (IgG) was used instead of anti-vinculin in some experiments to serve as negative controls. After incubation with the primary antibody, the cells were washed and incubated for 45 min with fluorescein isothiocyanate (FITC)-antimouse (1:200) and streptavidin-rhodamine (TRITC)-conjugated phalloidin (1:200; Chemicon International, Inc., Nutley, NJ) for 1 h. After additional washes, the cells were mounted using fluorescence mounting medium. (Vector Laboratories, Inc., Burlingame, CA) The staining pattern was visualized by a Zeiss 100M confocal microscope (Carl Zeiss Jena GmbH, Jena, Germany).

For qualitative evaluation of the actin cytoskeleton, a 40× or 60× objective was used during confocal imaging, and a z-series at 0.5- or 1.0-μm intervals was created. For each sample, 25 image fields were routinely photographed, and a total of 100 images were taken after four trials of the experiment. The number of CLANs in each sample was counted using Aequitas IDA software (version 1.3; DDL Ltd, Cambridge, UK). CLANs were defined as a structure with at least five hubs and three triangulated arrangements of spokes. The total number of cells in each image were counted by using nuclei staining. The severity of CLAN formation was presented as a ratio of CLAN number to cell number. Data were expressed in histogram form showing fold change to untreated NTM cells.

### Statistical analysis

All assays were performed using at least three separate experiments in triplicate, and data were expressed as mean±standard error (SE). A one-way analysis of variance (ANOVA) test was performed, and statistical significance was set at p<0.05.

## Results

### Dexamethasone does not change the morphology of trabecular meshwork cells

**Figure 1:**
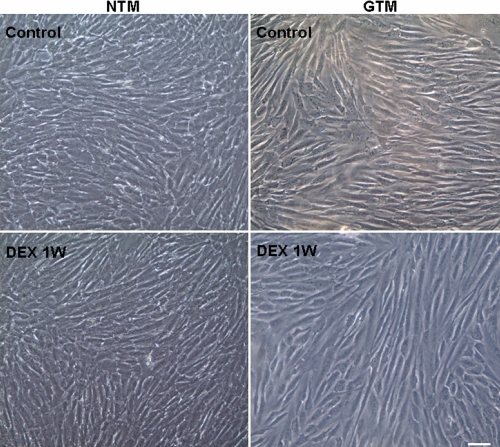
The morphology of trabecular meshwork cells before and after dexamethasone  treatment. Normal human trabecular meshwork (NTM) and primary open angle glaucoma trabecular meshwork (GTM) cells were obtained and cultured under identical culture protocol. GTM cells were slightly larger compared to NTM cells. Cell morphology shows no significant changes after treatment with 10^−7^ mol/l DEX for 1 week (1W) compared to the untreated control. Scale bar=50 μm.

As shown in Figure 1, both NTM and GTM cells reached complete confluency at 7 days. GTM cells appeared to be larger and more irregular in shape compared to NTM cells. TM cells incubated with DEX (10^−7^ M) for 7 days had no significant morphological changes, as observed using phase-contrast light microscopy (Figure 1).

### Trabecular meshwork cells express *ZO-1* and *Cx43*

**Figure 2:**
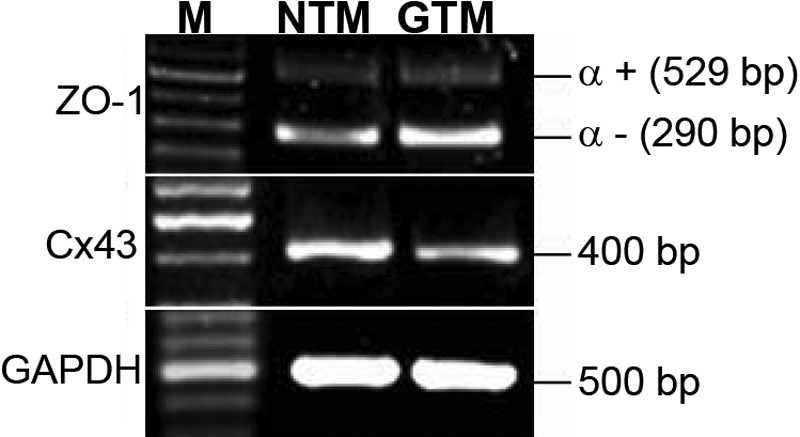
Trabecular meshwork cells express zonula occludens 1 and *Cx43*. NTM cells have lower zonula occludens 1 (*ZO-1*) α– isoform levels but higher connexin 43 (*Cx43*) levels compared to GTM cells, yet NTM and GTM cells have similar *ZO-1* α+ levels, as illustrated by RT–PCR. *GAPDH* was used as the internal loading control. M stands for molecular size ladder. n=3.

We next examined the expression levels of *ZO-1* and *Cx43* in the TM cells. Both NTM and GTM cells expressed ZO-1 isoforms and Cx43, as determined by RT–PCR (Figure 2). The ZO-1 α– isoform was more abundantly expressed compared to the α+ isoform in both NTM and GTM cells (Figure 2). GTM cells had higher levels of ZO-1 α– but lower *Cx43* mRNA levels compared to NTM cells (Figure 2). *GAPDH* was used as the internal loading control.

### Dexamethasone changes the expression of ZO-1 and Cx43 in cultured trabecular meshwork cells

**Figure 3:**
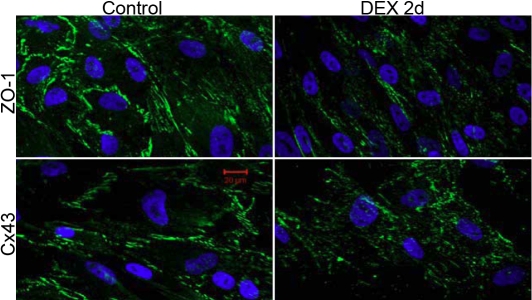
Effects of dexamethasone on zonula occludens 1 and connexin 43 in normal trabecular meshwork cells. Fixed normal trabecular meshwork (NTM) cells were immunolabeled with zonula occludens 1 (ZO-1) and connexin 43 (Cx43) (green fluorescence) and observed under a confocal microscope, using identical parameters. The ZO-1 antibody recognizes both α+ and α– isoforms of ZO-1. Both ZO-1 and Cx43 were plasma membrane bound. Treatment with 10^−7^ mol/l dexamethasone (DEX) for 2 days resulted in no significant changes in expression and distribution of ZO-1. However, DEX increased the cytoplasmic pool of Cx43. Nuclei are shown by DAPI staining (blue fluorescence). The scale bar=20 μm.

The immunocytochemistry study showed the plasma membrane localization of ZO-1 and Cx43 in NTM cells (Figure 3). Exposure to 10^−7^ M DEX for 2 days had no change in the expression level of ZO-1 and Cx43. However, DEX treatment increased cytoplasmic Cx43 but had no effect on ZO-1. This phenomenon may imply a problem assembling of the Cx43-positive gap junction (Figure 3).

**Figure 4:**
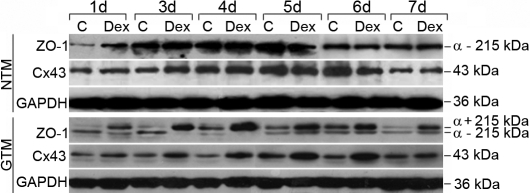
Dexamethasone increases zonula occludens 1 and connexin 43 expression in trabecular meshwork cells. Western blot analysis showed the expression of zonula occludens 1 (ZO-1) and connexin 43 (Cx43) protein in trabecular meshwork (TM) cells. NTM cells express the ZO-1 α– isoform and Cx43 protein, whereas GTM cells express both ZO-1 isoforms and Cx43 protein. Treatment with 10^−7^ mol/l  dexamethasone (Dex) caused a time-dependent increase in both ZO-1 and Cx43, with peak expression at 4 and 5 days, followed by a gentle decline at 6 days; however, the levels of these proteins were not at the basal level after 7 days. GAPDH was used as the internal loading control and n=3.

From western blot analysis, two ZO-1 isoforms were identified by a slight molecular weight shift since the α+ isoform is 80 amino acids longer than α– isoform. The expression of the ZO-1 α– isoform and the Cx43 protein were observed in both NTM and GTM cells, while the ZO-1 α+ isoform was unique to GTM cells (Figure 4). The levels of ZO-1 and Cx43 increased with the number of days in culture and peaked at around 4–5 days. This could be a result of junction formation with neighboring cells in the culture (Figure 4). DEX induces an increase in the ZO-1 α+ isoform specifically in GTM cells. Treatment with 10^−7^ M DEX caused a time-dependent increase in both ZO-1 and Cx43 (Figure 4). The peak expression of ZO-1 and Cx43 was observed at 4 days and 5 days, and then the expression levels declined at 7days but not to reach to the basal level (Figure 4). GAPDH was used as the internal loading control.

### Actin dysorganization in trabecular meshwork cells from patients, aggravated by dexamethasone

**Figure 5:**
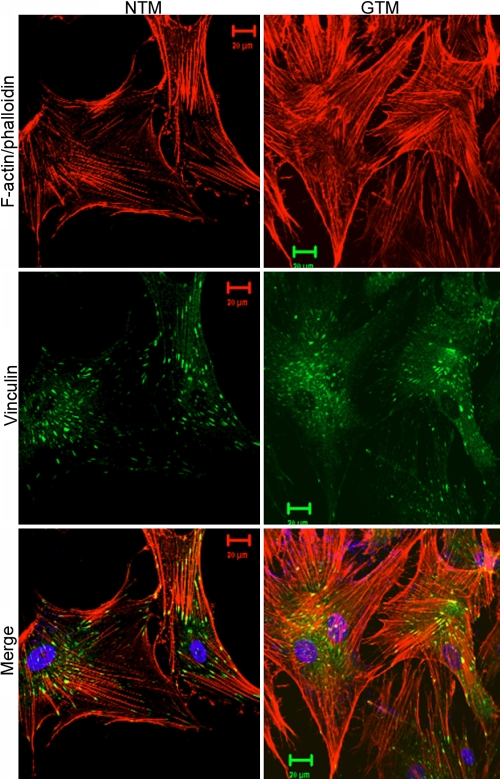
The cytoskeleton architecture of glaucomatous trabecular meshwork cells is tangled. The organization of the filamentous actin (F-actin; red fluorescence), and the distribution of vinculin (green fluorescence), the actin focal adhesion point on plasma membrane, were observed using confocal microscopy. The glaucomatous trabecular meshwork cells have a more irregular actin architecture and vinculin distribution compared to normal trabucular meshwork cells.

**Figure 6:**
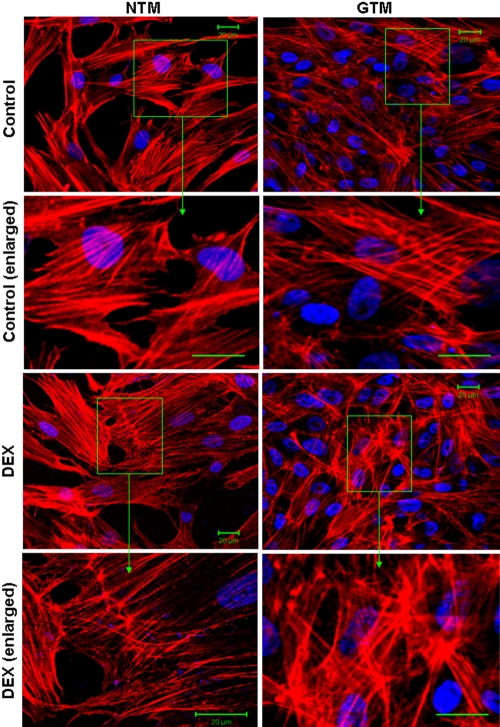
Treatment with 10^−7^ mol/l dexamethasone (DEX) for 7 days increased cross-linked actin networks formation in both normal trabecular meshwork and trabecular meshwork cells derived from POAG individuals cells. cross-linked actin networks (CLANs) were also observed in the untreated trabecular meshwork cells derived from POAG individuals (GTM) cells. Green squares indicate areas of enlargement. Nuclei were shown by DAPI staining (blue fluorescence).

**Figure 7:**
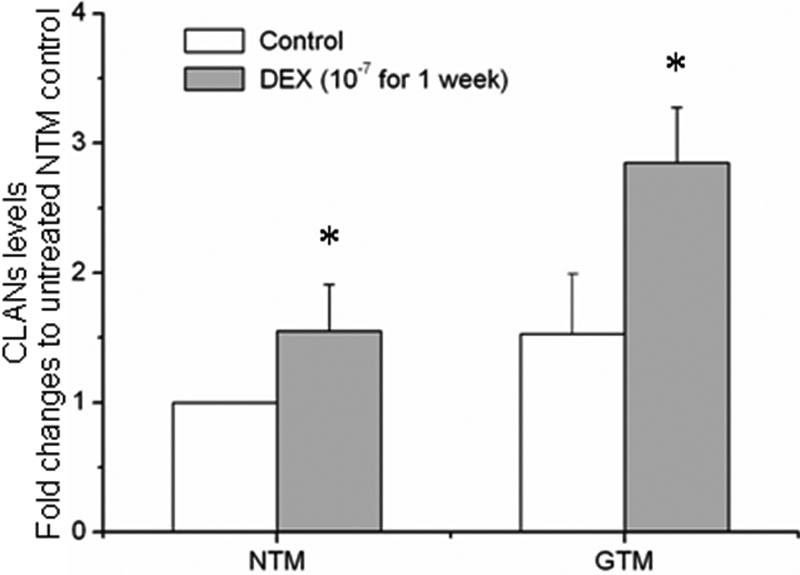
Histograms represent the fold changes in cross-linked actin networks to cell number ratios. The ratio for untreated NTM cells was arbitrarily set as 1. DEX caused a significant increase in cross-linked actin network (CLAN) numbers in both NTM and GTM cells compared to their respective untreated control. The asterisk indicates data significantly different from the respective untreated control at p≤0.05; the scale bar is 20 μm.

Figure 5 shows the distribution and organization of the F-actin cytoskeleton in cultured TM cells with or without DEX treatment. Both GTM and NTM cells are immunopositive to F-actin and vinculin (Figure 5). Although CLANs were observed in both NTM and GTM cells, the distribution of F-actin in GTM cells seems more irregular and tangled compared to NTM cells (Figure 6). DEX treatment increased overall actin staining in both NTM and GTM cells. The expanded view showed CLAN formation and serious actin tangling after DEX treatment (Figure 6). By counting CLANs in micrographs obtained from three separate experiments, DEX was found to cause 1.5-fold and 2.9-fold more CLANs in NTM and GTM, respectively (Figure 7).

## Discussion

GC-induced ocular hypertension and secondary open-angle glaucoma are serious adverse effects of GC therapy. Its clinical presentation is similar in many ways to POAG, including increased aqueous humor outflow resistance and morphological and biochemical changes to the TM. IOP elevation causes optic nerve damage and eventually leads to blindness in late stages. Here we investigated the effects of GCs on junctional protein and cytoskeleton organization in normal or glaucomatous TM cells based on observations from previous studies: (1) DEX treatment increases tight junction protein ZO-1 expression in both TM and Schlemm’s canal endothelial cells [2,24]; (2) ZO-1 has a direct interaction with Cx43, through which intercellular communication and barrier functions are mediated [25,26]; and (3) ZO-1 affects cell contractility via binding to cytoskeleton actin through anchoring protein α-actinin [27,28].

*ZO-1* has two splice variants, α+ and α–. The α+ isoform, which is the longer isoform, contains an additional 80 amino acids, is usually found in structurally less dynamic junctions, and is mostly detected in epithelial cells. In contrast, the α– isoform expresses in more dynamic junctions and is commonly found in endothelial cells [29,30]. During embryonic development, the α+ isoform appears right before formation of the nascent blastocele and tight junctions, whereas the α– isoform is present much earlier at the blastomere stage before tight junctions are assembled [31]. In agreement with the findings of Alvarado and colleagues that TM cells have less α+ protein relative to α– protein [24], we found an almost twofold less abundance of α+ mRNA levels compared to α– (Figure 3), indicating junctions of lower resistance may be found in NTM cells that allow more dynamic activities between cells. Interestingly, the α+ protein is too weak to be detected in NTM cells regardless of the treatment, but treatment with DEX induces a strong α+ signal in GTM cells, while the α– signal fades out in GTM cells (Figure 4). Studies on ZO-1 distribution suggest that the α+ isoform forms high-resistance junctions, while the α– isoform is present in relatively leaky adherens-type junctions [31]. This may explain the high aqueous outflow resistance in glaucomatous TM samples. In addition, since the α+ isoform forms a less active junction, the strong expression of α+ in GTM cells may indicate a loss of function change in the TM structure.

On the other hand, Cx43 is the most ubiquitously expressed vertebrate gap junction protein that is located on lateral plasma membrane to facilitate cell-cell communication [28,30]. The gap junctions allow both intercellular ionic and biochemical coupling to facilitate movement of ions (e.g., potassium) and small molecules (e.g., metabolites, sugars, lactate, and butyrate) as well as intracellular signaling molecules (e.g., cyclic nucleotides) [30,32]. Cx43 is found in both NTM and GTM cells. DEX increases the cytoplasmic pool of Cx43, indicating that the Cx43 protein fails to incorporate into gap junctions. The cellular aggregation of Cx43 may create a crowd effect and interfere with normal cellular functioning.

Apart from the junctional status, the cytoskeleton also controls the drainage rate of the TM. The distribution and organization of F-actin affects the contractility of the TM, in which contraction decreases and relaxation increases aqueous drainage [33,34]. Since the cytoskeleton also plays a role in maintaining cell polarity and orienting the metabolic and signal transduction machinery in cells, dysorganization of the cytoskeleton can greatly distort the functionality and integrity of TM cells [33-36]. In line with other observations, there are more CLANs in GTM and in cells exposed to DEX (Figure 6) [11,37]. CLANs are polygonal structures, and little is known about their functional significance and formation mechanisms. Atomic force measurements indicate that they seem to impart rigidity to the cell because the geodesic cross-linking arrangements are believed to make the TM cells “stiffer” and therefore more resistant to aqueous outflow [38,39]. Although we do not know whether CLANs are the initiator or the result of glaucoma, the current observations prompt us to hypothesize that suppression of CLAN formation or reversal of CLANs may be of therapeutic importance for POAG.

For example, latrunculin-A and -B, isolated from marine sponge, are a potent microfilament disruptor by sequestering actin. These compounds can prevent and reverse DEX-induced actin cytoskeleton re-organization as well as effectively increase the outflow facility when topically applied on nonhuman primate eyes [40] and postmortem human eyes [41]. Since latrunculin-A and other actin depolymerizing agents, such as cytohalasin D, can enhance matrix metalloprotease-2 activities to degrade and remodel ECM, and activate other cell survival molecules, including p38 mitogen-activated protein kinases (MAPK) and extracellular signal-regulated kinases (ERK1/2), in TM cells [42]. Thus, these agents are now under intensive investigation for development of future glaucoma therapy.
